# Clinical and Radiological Outcomes of Revision Anterior Cruciate Ligament Reconstruction Using a Quadriceps Tendon Autograft with a Bone Block: A Single-Center Case Series

**DOI:** 10.3390/medicina61091634

**Published:** 2025-09-10

**Authors:** Dhong Won Lee, Sung Gyu Moon, Ji Hee Kang, Seung Ik Cho, Woo Jong Kim

**Affiliations:** 1 Department of Orthopaedic Surgery, KonKuk University Medical Center, Konkuk University School of Medicine, Seoul 05030, Republic of Korea; 2 Department of Radiology, KonKuk University Medical Center, Konkuk University School of Medicine, Seoul 05030, Republic of Korea; sgmoon@kuh.ac.kr (S.G.M.); 20200184@kuh.ac.kr (J.H.K.); 3 Sports Medical Center, KonKuk University Medical Center, Konkuk University School of Medicine, Seoul 05030, Republic of Korea; 20150246@kuh.ac.kr; 4 Department of Orthopaedic Surgery, Soonchunhyang University Hospital Cheonan, Cheonan 31151, Republic of Korea; kwj9383@hanmail.net

**Keywords:** ACL, anterior cruciate ligament, quadriceps graft, QT autograft, revision ACL

## Abstract

*Background and Objectives:* Revision anterior cruciate ligament reconstruction (ACLR) is demanding and yields inferior outcomes compared with primary procedures. The quadriceps tendon (QT) autograft with bone block has biomechanical and biological advantages though clinical evidence in revision remains limited. This study evaluated the clinical and radiological outcomes of revision ACLR using bone-block QT autograft in young, active patients. *Materials and Methods:* A case series with a level of evidence of 4. Thirty-four patients (28 men, 6 women; mean age, 27.2 ± 5.8 years) who underwent revision ACLR with a bone-block QT autograft between 2021 and 2023 were retrospectively reviewed. The mean follow-up was 37.4 ± 3.2 months. Clinical assessments included the Lysholm, International Knee Documentation Committee (IKDC) subjective, and Tegner activity scores, along with isokinetic strength testing. Objective stability was evaluated using pivot shift grading and Telos stress radiography. Radiological analyses included 3D computed tomography for tunnel positioning and magnetic resonance imaging for tunnel widening. Perioperative and postoperative complications were recorded. *Results*: All clinical outcomes improved significantly from baseline to 2-year follow-up: Lysholm (62.7 ± 9.6 to 87.1 ± 10.3), IKDC (59.0 ± 10.8 to 79.5 ± 11.1), and Tegner (3.5 ± 1.2 to 5.6 ± 1.3; all *p* < 0.001). However, the Tegner score remained lower than the pre-injury level (6.1 ± 1.4; *p* = 0.035). At the final follow-up, 91.2% of the patients had returned to sports, with 59% resuming sports at their pre-injury level or higher. Side-to-side anterior laxity decreased from 8.5 ± 1.7 mm to 1.4 ± 1.1 mm on Telos stress radiography (*p* < 0.001). Preoperatively, 82% of patients demonstrated high grade pivot shift (≥grade 2), which improved to 91% graded as negative or grade 1 at final follow-up (*p* < 0.001). Isokinetic evaluation showed improvements in quadriceps (28.7% ± 12.5% to 12.4% ± 8.1%) and hamstring (18.3% ± 9.7% to 8.9% ± 6.5%) deficit (both *p* < 0.001). MRI demonstrated minimal tunnel widening (tibia, +1.3 ± 0.9 mm, *p* = 0.012; femur, +0.3 ± 0.6 mm, *p* = 0.148). Three complications (8.8%) were observed: one cyclops lesion, one transient extension deficit, and one graft rupture. No patellar fractures, septic arthritis, or revision procedures occurred during the follow-up period. *Conclusions*: Bone-block QT autografts provide a reliable option for revision ACLR, yielding functional improvement, restored stability, and minimal donor-site morbidity, with low complications. These findings support their consideration as the preferred graft choice for young active patients needing revision reconstruction.

## 1. Introduction

Anterior cruciate ligament reconstruction (ACLR) is the standard surgical intervention for restoring knee stability, reestablishing anterior cruciate ligament function, and facilitating return to sports activity [1,2,3]. Despite advances in surgical techniques and rehabilitation, revision ACLR is occasionally required due to structural failure of the graft, new traumatic events, or suboptimal biological incorporation [4,5,6,7]. Compared with primary ACLR, revision ACLR presents substantially greater technical challenges and is associated with inferior outcomes in terms of patient-reported outcome measures, objective stability, return-to-sport rates, and graft survival [8,9,10]. These suboptimal outcomes are multifactorial, reflecting structural alterations from prior surgery (e.g., tunnel widening and bone loss), residual rotatory or varus instability due to concomitant anterolateral or posterolateral corner insufficiency, and associated meniscal or chondral lesions [8,9,10]. Previous reviews have consistently reported that revision ACLR is associated with higher rates of concomitant lesions and increased surgical complexity, particularly in young, highly active patients for whom graft choice becomes a critical determinant of surgical success [11,12].

In young and highly active patients, the increased risk of re-injury and functional decline underscores the importance of appropriate graft selection [13]. Among the available autograft options, the bone-patellar tendon-bone (BPTB) graft has long been valued for its strong fixation and bone-to-bone healing; however, donor-site morbidity, including anterior knee pain, harvest-site discomfort, and contralateral knee symptoms, remains a concern [14]. Hamstring tendon autografts are associated with lower donor-site morbidity and smaller incisions; nonetheless, they may provide an insufficient graft diameter and are frequently unavailable in the revision setting [14,15]. Allografts eliminate donor-site morbidity and shorten the operative time; however, they have been associated with delayed biological incorporation and higher failure rates in younger and more active populations [16].

The quadriceps tendon (QT) autograft with a bone block has gained attention as a versatile alternative to revision ACLR [17,18]. It offers a broad cross-sectional area, high collagen density, predictable graft size, and secure fixation through bone-to-bone healing when harvested using a bone block [17,18]. Importantly, QT autografts can be obtained from patients with prior BPTB or hamstring harvest, thereby expanding the graft options in complex revision cases [18]. Recent meta-analyses and comparative studies suggest that QT autografts in revision ACLR achieve stability and functional outcomes comparable or superior to those of BPTB or hamstring autografts while maintaining lower donor-site morbidity [5]. However, evidence of the short- to mid-term clinical and radiological outcomes of bone-block QT autografts in the revision setting remains limited.

Therefore, this study aimed to evaluate the clinical and radiological outcomes of revision ACLR using a bone-block QT autograft and to assess its utility as a graft choice in technically demanding revision scenarios. We hypothesized that bone block QT autografts would yield favorable knee stability and functional outcomes with a low complication rate in this patient population.

## 2. Materials and Methods

### 2.1. Patient Selection

This retrospective case series reviewed 39 patients who underwent revision ACLR using a QT autograft with a bone block between 2021 and 2023. The study protocol was approved by our Institutional Review Board (KUMC 2025-08-046). The inclusion criteria were: aged between 18 and 50 years, confirmed failure of primary ACLR requiring revision, and the availability of complete medical records, together with both preoperative and postoperative clinical evaluations and radiological studies. The exclusion criteria were advanced osteoarthritis of the ipsilateral knee (Kellgren-Lawrence grade ≥ 3), a history of ligament reconstruction or cartilage restoration procedures on the contralateral knee, multi ligament injuries requiring concurrent reconstruction other than anterolateral ligament augmentation, and a follow up period of less than 24 months. Based on these criteria, five patients were excluded from the study. Three patients had a history of contralateral ACLR, and two did not complete both the clinical and radiological evaluations by the two-year follow-up. The final study cohort comprised 34 patients.

### 2.2. Surgical Techniques

The decision between 1-stage and 2-stage revision ACLR was made preoperatively based on standard radiographs and computed tomography (CT). A 1-stage revision was performed when the previous tunnels were anatomical or non-anatomical without significant overlap, with tunnel diameters of <12 mm and adequate bone stock. A 2-stage revision was indicated for tunnel malposition with risk of convergence, tunnel enlargement ≥12 mm, or osteolytic changes compromising fixation [7]. In the 2-stage protocol, the first stage involves hardware removal and bone grafting to fill the widened tunnels and restore adequate bone stock. The second stage was performed approximately six months later, after CT confirmed complete bone graft incorporation.

**Figure 1:**
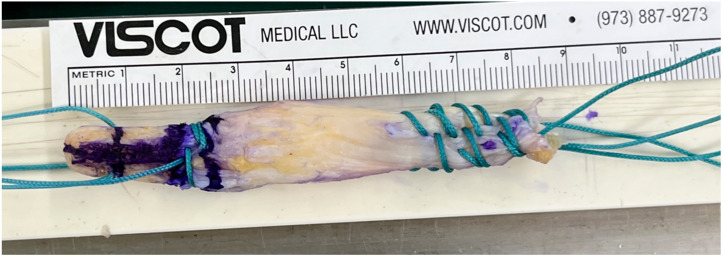
Gross photo of a quadriceps tendon autograft with a bone block.

A QT autograft with a bone block was harvested through a minimal transverse incision along the superior border of the patella, using a tendon harvester system (Karl Storz, Tuttlingen, Germany). The central tendon strip was dissected to approximately 10 mm in width and 70–75 mm in length. A bone block measuring 10 mm in diameter and 15–20 mm in length was obtained from the superior pole of the patella to facilitate bone-to-bone healing. Both ends of the graft were prepared using nonabsorbable sutures, and the bone block was contoured to match the femoral tunnel diameter (Figure 1).

**Figure 2:**
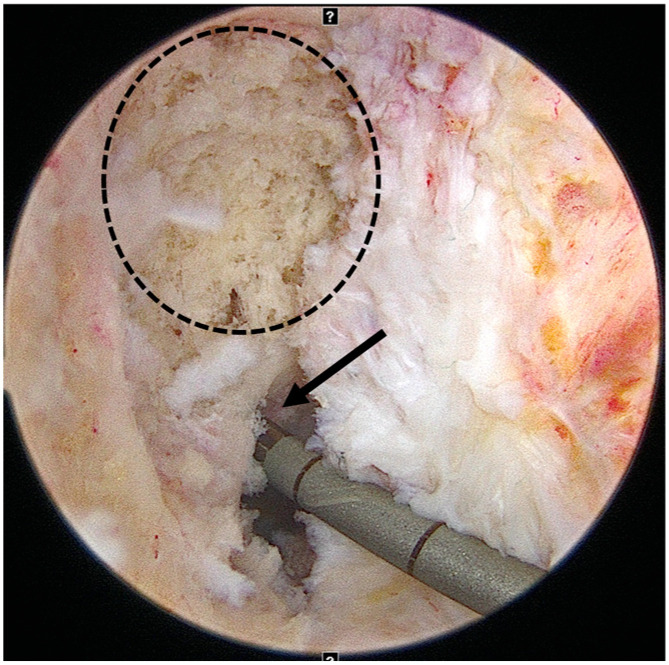
One-stage revision anterior cruciate ligament reconstruction (ACLR). The dotted circle indicates the previous nonanatomic femoral tunnel, which was bone grafted with an allograft. The arrow demonstrates creation of a new anatomic femoral tunnel using a flexible reamer system.

**Figure 3:**
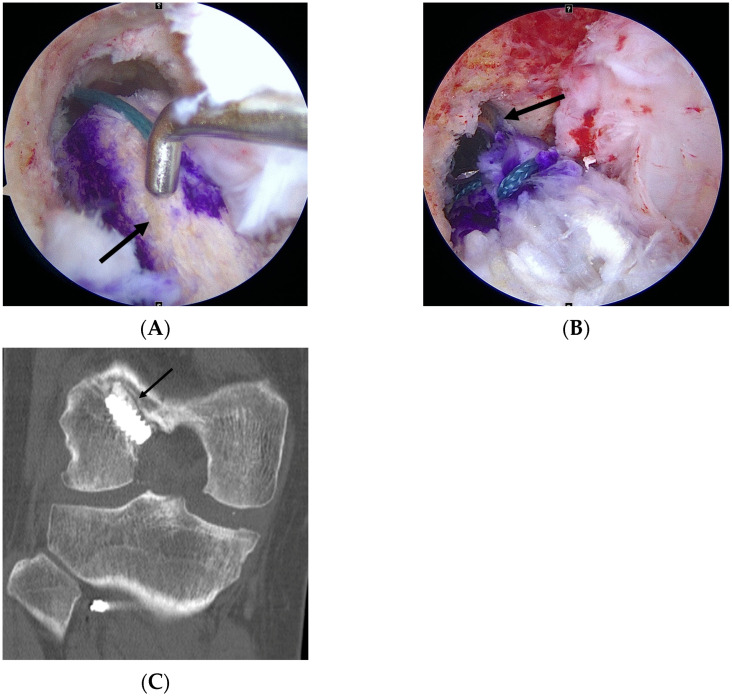
Femoral tunnel bone–block fixation. (**A**) The bone block (black arrow) is carefully introduced into the femoral tunnel, with a probe used to guide insertion and maintain proper orientation. (**B**) Intraoperative view showing the bone block fully seated within the tunnel and stabilized by a metallic interference screw (black arrow). (**C**) Postoperative coronal CT image demonstrating secure fixation of the bone block (black arrow) with the metallic interference screw within the femoral tunnel.

The femoral tunnel was created at the anatomical center using a flexible reamer system (Stryker, Kalamazoo, MI, USA) consistent with the technique previously reported by Lee et al. [19] (Figure 2). The tibial tunnel was prepared at the anatomical center along the posterior border of the lateral meniscal anterior horn. Both tunnel diameters were precisely matched to the graft size. The bone block was positioned in the femoral tunnel and fixed with a metallic interference screw to achieve bone-to-bone fixation (Figure 3). On the tibial side, the tendon portion was fixed using a bioabsorbable interference screw supplemented with a post-tie, if necessary. Graft tensioning was performed with the knee at 20° of flexion before the final fixation.

Anterolateral ligament reconstruction was performed in all patients using a tibialis allograft. The femoral attachment was located proximal and posterior to the lateral epicondyle, and the tibial attachment was placed midway between Gerdy’s tubercle and the fibular head. Fixation at both ends was achieved using bioabsorbable interference screws, tensioning the graft at 20° of knee flexion in neutral rotation.

### 2.3. Postoperative Rehabilitation

All patients underwent a standardized rehabilitation protocol supervised at our institution’s sports medicine center. Patients were evaluated and instructed at 3 and 6 weeks, 12 weeks, and subsequently every 3 months until 1 year postoperatively. At each visit, structured assessments and stage-specific education were provided to ensure adherence and progressive recovery. An ACL brace (SL Knee–K001, Seoul Brace, Seoul, Republic of Korea) was worn for 3 months. For the first 3 weeks, range of motion (ROM) was limited to 0° to protect the graft and allow early biological healing. The ROM was gradually increased to 90° by 6 weeks and 120° by 12 weeks. Partial weight-bearing with crutches was permitted immediately postoperatively and progressed to full weight-bearing at 6 weeks.

Quadriceps setting and straight leg-raising exercises were initiated within the first postoperative week to prevent muscle atrophy. Six weeks postoperatively, the patients began closed kinetic chain strengthening, stationary cycling, and proprioceptive training using a balance board. At 4–5 months, light jogging was introduced if the quadriceps strength was ≥70% of the contralateral limb, as measured by isokinetic dynamometry. Return to noncontact sports was permitted after 9 months, and full return to pivoting sports was allowed no earlier than 12 months postoperatively, provided quadriceps and hamstring strength was ≥90% limb symmetry, and clinical stability was confirmed by physical examination.

### 2.4. Clinical Evaluation

All patients were evaluated preoperatively and at 2 years postoperatively. Patient-reported outcomes included the Lysholm knee score, Tegner activity scale, and International Knee Documentation Committee (IKDC) subjective scores. Objective assessments consisted of pivot-shift tests, and isokinetic quadriceps and hamstring strength testing (Biodex Medical Systems, Shirley, NY, USA) at angular velocities of 60°/s. Peak torque deficits were expressed as percentages relative to the contralateral limb. Return-to-sport status at the final follow-up was recorded and classified as return to preinjury level, return to lower level, or no return to sport. Perioperative and postoperative complications, including graft re-rupture, infection, stiffness, and hardware-related symptoms, were also documented.

### 2.5. Radiological Evaluation

**Figure 4:**
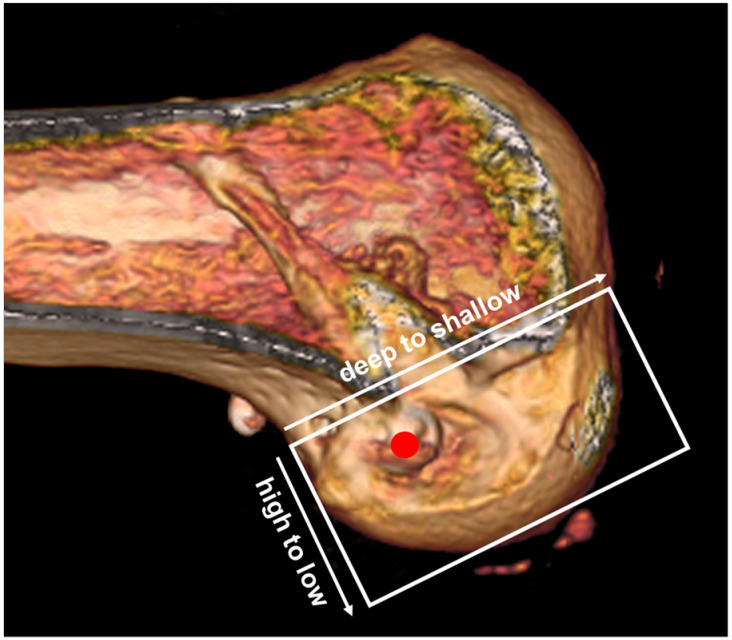
Femoral tunnel placement by the quadrant method. The red dot indicates the center of the anatomical femoral tunnel.

Radiological assessment included Telos stress radiography (Telos Medical Instruments, Marburg, Germany) performed preoperatively and 2 years postoperatively, applying a standardized 134-N anterior load at 20° of knee flexion to measure side-to-side anterior tibial translation. Three-dimensional CT scans (LightSpeed VCT XT; GE Medical Systems, Milwaukee, WI, USA) were obtained 2 days postoperatively to evaluate the femoral and tibial tunnel positions in accordance with the quadrant method for the femur and the Amis method for the tibia [20] (Figure 4). The same CT protocol was repeated at 2 years. Tunnel diameters were measured perpendicular to the tunnel axis at three predefined levels—aperture, mid-portion, and exit—on axial, sagittal, and coronal planes. For each location, the largest diameter was recorded, and the difference between baseline (day 2) and follow-up (2 years) values was defined as the degree of tunnel widening [21]. All measurements were independently performed by an experienced orthopedic surgeon (D-W.L.) and a musculoskeletal radiologist (S-G.M.), both blinded to the clinical outcomes. The mean of the two measurements was used for the analysis.

### 2.6. Statistical Analysis

All statistical analyses were performed using the SPSS software (version 25.0; IBM Corp., Armonk, NY, USA). Continuous variables are presented as means with standard deviations and categorical variables as counts with percentages. Normality of the data distribution was assessed using the Shapiro–Wilk test. Paired *t*-tests were used to compare pre-and postoperative continuous variables for normally distributed data, whereas the Wilcoxon signed-rank test was used for non-parametric data. Categorical variables were compared using the chi-square test or Fisher’s exact test, as appropriate. Intra- and inter-observer reliabilities for radiologic measurements were assessed using the intraclass correlation coefficient (ICC). Statistical significance was set at *p* < 0.05.

## 3. Results

A total of 34 patients (28 males, 6 females; mean age, 27.2 ± 5.8 years) met the inclusion criteria and were available for the final analysis. The basic demographics of enrolled patients are summarized in Table 1. The mean follow-up duration was 37.4 ± 3.2 months. Twenty-six patients (76.5%) underwent single-stage revision ACLR, while 8 (23.5%) underwent two-stage procedures. In all two-stage cases, the interval between first-stage bone grafting and second-stage revision was 6 months. Concomitant procedures included meniscal repair in 9 patients (26.5%), partial meniscectomy in 7 (20.6%), and cartilage procedures in 4 (11.8%).

All clinical scores significantly improved from the preoperative assessment to the 2-year follow-up (Table 2). The mean Lysholm score increased from 62.7 ± 9.6 preoperatively to 87.1 ± 10.3 at 2 years (*p* < 0.001). The IKDC subjective score improved from 59.0 ± 10.8 to 79.5 ± 11.1 (*p* < 0.001). On the Tegner activity scale, patients reported a pre-injury level of 6.1 ± 1.4, which declined markedly to 3.5 ± 1.2 prior to surgery. At 2 years after revision ACLR, activity level improved to 5.6 ± 1.3, representing a significant gain compared with the preoperative score (*p* < 0.001), although it remained lower than the pre-injury level (*p* = 0.035). At the final follow-up, 31 patients (91.2%) had returned to sports and 20 (59%) resumed participation at their pre-injury level or higher. Preoperatively, 25 patients (73.5%) demonstrated a high-grade pivot-shift (grade ≥ 2), whereas at 2 years, 31 patients (91.2%) showed grade 0–1 stability, with no patient exhibiting grade 3 rotational instability (*p* < 0.001) (Table 2). Isokinetic testing at 60°/s demonstrated significant recovery of muscle function. The quadriceps peak torque deficit decreased from 28.7% ± 12.5% preoperatively to 12.4% ± 8.1% at 2 years (*p* < 0.001). Similarly, the hamstring peak torque deficit improved from 18.3% ± 9.7% to 8.9% ± 6.5% (*p* < 0.001) (Table 2).

Stress radiographs obtained using the Telos device with an anterior load of 134 N demonstrated a reduction in mean side-to-side difference from 8.5 ± 1.7 mm preoperatively to 1.4 ± 1.1 mm at 2 years (*p* < 0.001).

**Figure 5:**
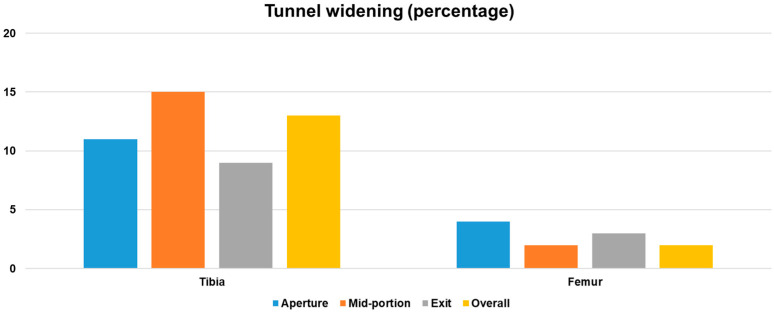
Bar diagram showing tunnel widening at three predefined levels (aperture, mid-portion, exit) in the tibial and femoral tunnels at 2 years postoperatively. Values represent mean changes in tunnel diameter, with each level determined by the largest measurement among axial, sagittal, and coronal CT planes relative to baseline measurements at postoperative day 2.

Three-dimensional CT scans obtained 2 days postoperatively confirmed the anatomic positioning of both the femoral and tibial tunnels within the recommended ranges, based on the quadrant method for the femur and the Amis method for the tibia. The mean femoral tunnel position was 30.6% ± 3.2% along the Blumensaat line (deep–shallow axis) and 26.9% ± 2.8% along the notch height (high–low axis). The mean tibial tunnel position was 42.3% ± 3.5% in the anteroposterior direction and 48.7% ± 3.8% mediolaterally. At the 2-year follow-up, tibial tunnel widening was observed at all measured levels compared with baseline CT obtained on postoperative day 2. Enlargement was 11% at the aperture (*p* = 0.021), 15% at the mid-portion (*p* < 0.001), and 9% at the exit (*p* = 0.043). The mid-portion demonstrated the greatest relative increase among the three levels. When averaged across all levels and planes, the tibial tunnel widened by 13% overall, which reached statistical significance (*p* = 0.012). In contrast, femoral tunnel changes were negligible. The relative increases were 4% at the aperture (*p* = 0.162), 2% at the mid-portion (*p* = 0.274), and 3% at the exit (*p* = 0.188), yielding an overall mean widening of 3%, which did not reach statistical significance (*p* = 0.148) (Figure 5). Intraobserver and interobserver ICC values for tunnel measurements were 0.93 (95% CI, 0.88–0.96) and 0.90 (95% CI, 0.85–0.94), respectively, indicating excellent reliability.

Postoperative MRI performed at 2 days and at 2 years were used to assess tunnel widening. The initial tunnel diameter was standardized at 10.0 mm for both the femoral and tibial tunnels. At final follow-up, the mean tibial tunnel diameter increased by 1.3 ± 0.9 mm (*p* = 0.012), Femoral tunnel enlargement was minimal, with a mean increase of 0.3 ± 0.6 mm, which did not reach significance (*p* = 0.148).

Postoperative complications occurred in 3 patients (8.8%). These included 1 case of cyclops lesion requiring arthroscopic debridement, 1 case of persistent extension deficit that resolved with intensive physiotherapy, and 1 case of graft rupture. No cases of patellar fracture, septic arthritis, or residual instability requiring surgical intervention were observed during follow-up.

## 4. Discussion

The principal finding of this study was that revision ACLR using bone-block QT autografts led to significant improvements in patient-reported outcomes, objective knee stability, muscle strength, and return to sports within a mean follow-up of 27.4 months. These outcomes are notable given the technical demands and traditionally inferior prognosis of revision ACLR compared with primary procedures.

Previous studies have consistently highlighted the graft choice as a key determinant of stability in revision ACLR. Particularly, grafts with bone-to-bone healing, such as bone–patellar tendon–bone (BPTB) or QT, have shown a reproducible trend toward superior stability compared with soft-tissue hamstring autografts or allografts [22,23,24]. Consistent with the principle of bone-to-bone integration, femoral tunnel widening was negligible in our cohort, in contrast to prior reports with soft-tissue grafts where femoral tunnels frequently exhibited more pronounced enlargement [25]. Although tibial tunnel widening was observed, most apparent at the mid-portion, but the mean increase was modest (1.3 mm overall) and did not result in clinical compromise [25,26]. This distinction further underscores the biological and mechanical advantages of bone-block autografts in revision ACLR. In a meta-analysis of 606 revision ACLRs, QT and BPTB demonstrated lower failure rates (7.6% and 8.7%) than hamstrings (13.3%), and the mean side-to-side difference in arthrometric measurements favored QT (1.7 ± 0.6 mm) over hamstrings (2.1 ± 0.5 mm) [5]. A more recent systematic review including 859 patients substantiates this trend [27]. In that analysis, hamstring grafts were consistently associated with greater anterior laxity and a higher frequency of grade ≥ 2 pivot shifts, with rates reported in the range of 18–22%. Contrastingly, bone-block grafts demonstrated lower rates of approximately 9–13% for BPTB and 10–12% for QT. Although not every study reached significance, the overall synthesis of the data revealed a reproducible pattern. Overall, the pooled evidence indicates that, although not all comparisons reach statistical significance, the cumulative evidence consistently trends toward superior anterior stability and pivot-shift control when bone block grafts (BPTB, QT) are used in revision ACLR. A recent review comparing QT harvest with and without a bone block showed that bone-block QT achieved a mean side-to-side differences of 1.5–1.7 mm and high-grade pivot shift of <10%, compared with >20% when QT was used without a bone block [6].

Based on these findings, QT grafts have recently gained attention as alternative bone block options [17]. Compared with BPTB, QT offers a larger cross-sectional area, which may translate into superior load distribution and graft strength. Additionally, QT harvesting reduces donor-site morbidity such as anterior knee pain or kneeling discomfort, making it a biomechanically advantageous and clinically attractive choice for both primary and revision ACLR [18]. Even when the same bone-to-bone fixation principle is applied, the clinical outcomes appear to diverge depending on whether the graft is autologous or allogeneic. A recent single-institution series using quadriceps tendon-patellar bone allografts reported a mean residual side-to-side difference of 2.6 mm, with 92% of patients demonstrating a grade 0–1 pivot shift, but a re-rupture rate of 10.5% at a mean of 18 months [16]. Contrastingly, in our autograft QT cohort, the mean Telos side-to-side difference was only 1.4 mm, with 91% of the patients demonstrating a grade 0–1 pivot shift and a re-rupture rate of <5%. While a direct comparison is limited by the study design, these findings suggest that the superior biological viability of autografts may enhance the mechanical benefits of bone block fixation, resulting in improved stability and a reduced risk of graft failure in the revision setting.

BPTB has traditionally been considered the benchmark graft because of its reliable bone-to-bone healing. In the revision setting, however, available evidence does not demonstrate any clear advantage of BPTB over QT in terms of stability or graft survival. Moreover, BPTB harvest is often precluded if it was used in the index procedure and is associated with a higher incidence of anterior knee and kneeling pain. QT, in contrast, provides comparable or lower failure rates with fewer donor-site problems and can be reliably obtained in patients who have already undergone previous graft harvest. These observations, reinforced by recent systematic reviews and multicenter series, explain the increasing preference for QT as an autograft in revision ACLR [5,6,18,22]. Nevertheless, in the rare event of graft re-rupture after ACLR using autograft QT, alternative options such as a contralateral BPTB autograft or bone-block allografts (QT or BPTB) may be considered, depending on graft availability and patient-specific factors.

Returning to sports is another critical outcome after revision ACLR. Previous studies reported relatively low rates. In one single-center cohort, the overall return to sports rate was 71.7%, with only 12.1% returning to pre-injury levels [28]. Lee et al. [29] found that although 88% of patients resumed sports after revision, only 25.6% of isolated revisions regained their prior level compared with 57.1% when combined with anterolateral ligament reconstruction. A systematic review emphasized the particular vulnerability of female athletes, with overall return to sports rates of 50–60% and less than 40% achieving the pre-injury level [30]. This study achieved > 90% overall return to sports, with nearly 60% resuming or surpassing their pre-injury levels. These favorable outcomes not only exceed historical benchmarks, but also approach the best-performing subgroups in prior reports. Such results may reflect the enhanced stability of the quadriceps tendon autograft, adherence to structured rehabilitation, and careful patient selection, underscoring the fact that excellent recovery and sports participation remain achievable even in the revision setting. In addition, concomitant anterolateral ligament reconstruction likely contributed to improved rotational stability, reducing the risk of recurrent pivoting episodes, and further reinforcing patients’ confidence in returning to sports [29].

Donor-site morbidity and quadriceps weakness have been recognized as potential limitations of QT harvesting. Hughes et al. [31] demonstrated significant early extensor deficits with quadriceps limb symmetry indices (LSI) ranging from 69.5% to 83.3% between 5 and 15 months, underscoring the risk of residual weakness in the first postoperative year. Contrastingly, Horteur et al. [32] found no clinically meaningful deficits, with quadriceps strength symmetry largely restored after the early recovery phase. Extensor strength deficits did not significantly differ between the QT and hamstring groups under any test condition, with mean peak torque deficits of 33.1% in the QT group and 28.2% in the hamstring group at 60°/s during the first postoperative year (*p* = 0.42). Notably, flexor weakness was more pronounced in the hamstring muscle group, particularly at higher angular velocities corresponding to explosive contractions (14% vs. 3% at 240°/s, *p* = 0.04), underscoring the relative preservation of donor site function following QT harvest. Setliff et al. [33] prospectively followed patients for an average of 30.9 months (range, 24–48 months) and found that QT harvesting was not associated with clinically meaningful persistent donor-site morbidities. Time to 80% quadriceps strength did not differ significantly between groups (QT 131.7 ± 40.1 days vs. HS 149.5 ± 38.2 days, n.s.), indicating that recovery of extensor function after QT harvest was at least comparable to hamstring autografts. Importantly, patient-reported outcomes remained favorable, confirming reliable long-term recovery without residual extensor deficits. Our results are consistent with this recovery trajectory. Although the patients exhibited marked quadriceps weakness at baseline, isokinetic testing confirmed full recovery by 2 years post-operation with no residual strength deficits or functional complaints. Importantly, our minimally invasive harvesting technique using a specialized device (limiting the bone plug to <2 cm), combined with a structured rehabilitation program, likely facilitated this outcome. Consistent with both the available evidence and our own experience, we regard the bone-block quadriceps tendon autograft as the preferred graft for revision ACLR, owing to its predictable graft size, secure bone-to-bone fixation, and favorable donor-site profile compared with other autografts.

This study has several limitations. First, this was a retrospective case series, and the relatively small number of patients reflected the rarity of revision ACLR. Second, the lack of a control group prevented a direct comparison with alternative graft choices, highlighting the need for future comparative studies. Third, the study population was heterogeneous, including patients who underwent two-stage reconstructions and concomitant procedures, such as meniscal or cartilage surgery, which could have influenced the clinical outcomes. Finally, the average follow-up period of just over 2 years was relatively short, restricting conclusions regarding long-term graft survival or osteoarthritic changes, and longer follow-up will be required to confirm whether the favorable outcomes with quadriceps tendon autografts are maintained and to clarify their effect on graft durability and the risk of osteoarthritis in revision ACLR. Nevertheless, the unique strength of this study lies in its focus on revising ACLR using QT autografts, a setting for which there is limited prior evidence. By providing detailed data on quadriceps recovery timelines, demonstrating favorable patient-reported outcomes, and confirming the absence of clinically meaningful donor-site morbidity over a short-term follow-up period, this study adds important and novel evidence to the growing body of literature supporting QT as a viable option for revision reconstruction.

## 5. Conclusions

Bone-block QT autografts provide a reliable and versatile option for revision ACLR, yielding significant functional improvement, restored stability, and minimal donor-site morbidity, with a low complication rate. These findings support their consideration as the preferred graft choice for young active patients requiring revision reconstruction.

## Figures and Tables

**Table 1:** Demographic data.

	N = 34
Age, years	27.2 ± 5.8
Sex, n, Male/Female	28/6
Body mass index, kg/m^2^	23.6 ± 4.7
Follow-up duration, months	37.4 ± 3.2
Concomitant procedure, n (%)	
Meniscal repair	9 (26.5%)
Meniscectomy	7 (20.6%)
Cartilage repair	4 (11.8%)

**Table 2:** Comparison of Preoperative and Postoperative Outcomes.

	Preoperative	Last Follow-Up	*p*-Value
Lysholm score	62.7 ± 9.6	87.1 ± 10.3	<0.001
IKDC subjective score	59.0 ± 10.8	79.5 ± 11.1	<0.001
Tegner activity scale	3.5 ± 1.2	5.6 ± 1.3	<0.001
Pivot shift test (0/1/2/3), n	(0/9/10/15)	(29/2/3/0)	<0.001
Deficit of isokinetic extensor strength (%)	28.7 ± 12.5	12.4 ± 8.1	<0.001
Deficit of isokinetic flexor strength (%)	18.3% ± 9.7	8.9% ± 6.5	<0.001

## Data Availability

The data presented in this study are available upon request from the corresponding author.
